# Relation between the stability of dental implants and two biological markers during the healing period: a prospective clinical study

**DOI:** 10.1186/s40729-016-0058-y

**Published:** 2016-12-08

**Authors:** Choknapa Tirachaimongkol, Peraphan Pothacharoen, Peter A. Reichart, Pathawee Khongkhunthian

**Affiliations:** 1 Center of Excellence for Dental Implantology, Faculty of Dentistry, Chiang Mai University, Suthep sub-district, A. Muang, Chiang Mai 50200 Thailand; 2 Department of Biochemistry, Faculty of Medicine, Thailand Excellence Center for Tissue Engineering and Stem Cells, Chiang Mai, Thailand; 3 Department of Oral Medicine, Dental Radiology and Oral Surgery, Center for Dental, Oral and Maxillary Medicine, Charite – University of Medicine, Berlin, Germany

**Keywords:** Biomarker, Osseointegration, Implant stability quotient, Resonance frequency analysis

## Abstract

**Objectives:**

The purposes of this study were to examine the correlation between the stability of dental implants and bone formation markers during the healing period and to monitor the stability of dental implants using the resonance frequency analysis (RFA) method. The null hypothesis of the study is no correlation between the stability of dental implant and bone formation markers.

**Methods:**

The study is a prospective clinical study during the 3-month healing period of implant. At implant placement (PW Plus, Nakhon Pathom, Thailand) and after 1, 2, 3, 4, 6, 8, 10, and 12 weeks, RFA assessments were performed and gingival (GCF)/peri-implant crevicular fluids (PICF) were collected from ten patients. The level of osteocalcin (OC) was measured by using ELISA kits, and the level of alkaline phosphatase (ALP) activity was measured by colorimetric analysis. Repeated measures analysis of variance, the Friedman test, the Mann-Whitney *U* test, and the Pearson correlation were performed for data analysis.

**Results:**

There was a statistical decrease in the mean implant stability quotient (ISQ) values between 1 and 3 weeks (*P* < 0.05). The ISQ values recovered to the initial values at 4 weeks. There was no statistical difference in the ALP level at each measurement, while there was a statistical increase in the OC level at 6, 8, 10, and 12 weeks when compared with 1 week (*P* < 0.05). There was a significant correlation between ALP levels and ISQ values (*r* = 0.226, *P* < 0.05). There was a statistically significant correlation between OC levels and ISQ values at 1–12 weeks (*r* = 0.245, *P* < 0.05).

**Conclusions:**

The ISQ values were weakly correlated with both ALP and OC. The three-thread-design implant showed a high stability through healing period.

## Background

Dental implants have shown a high success rate for rehabilitation of edentulous patients if certain conditions are met during treatment. Nevertheless, the risk of failure remains difficult to predict. The achievement of osseointegration depends on many factors, such as a suitable host, biocompatible materials, careful surgery, and an appropriate healing time [1].

The primary stability comes from the mechanical anchorage between the bone tissue and the pitch region of the implant immediately after implantation. The secondary stability comes from the formation of new vital bone, which replaces the gap between the local bone and the implant surface and replaces the necrotic bone.

The external morphology of the dental implant is a factor which leads to the mechanical engagement of the implant with the bone. Three-thread-design dental implants consist of three different thread designs. The first design is the micro-thread, or supra-fine thread, which is on the coronal third of the implant fixture and is attached to the cortical bone. These small, fine threads are designed for force distribution, an increase in the bone-implant contact area and a decrease in the force concentration at the abutment-implant connection area. The second design is the reverse buttress thread, which is on the middle third of the implant fixture. This design increases the retention between the implant and spongy bone and produces resistance to compressive force. The third design, the condensed thread, is located at the apical third of the implant fixture. The thinness at the beginning of this thread and an increase in thickness along the implant fixture are designed for the soft spongy bone condensability [2].

Osseointegration, a continuous process, represents the coupling of the osteoclast and osteoblast activity for bone repair, formation, and adaptation to function [3, 4]. Implant-bone integration is separated into three phenomena. The first phenomenon is distance osteogenesis. Distance osteogenesis means that bone formation takes place from the local bone toward the implant surface. This event is anticipated to happen in cortical bone healing [5]. The second phenomenon is contact osteogenesis, in which bone formation takes place from the implant surface toward the local bone. This osteogenesis consists of the early phase of osteogenic cell migration, osteoconduction, and de novo bone formation. The de novo bone formation at a solid surface has four stages. The first stage is secretion of the two noncollagenous proteins, osteopontin, and bone sialoprotein. The second stage is calcium phosphate nucleation, which consists of the calcium binding at the calcium binding sites of these proteins. The third stage is the crystal growth phase. The last stage of de novo bone formation is derived from collagen production and subsequent collagen mineralization. Finally, bone remodeling is the third phenomenon of implant-bone integration [6].

Osteocalcin (OC) is the most plentiful noncollagenous protein of the bone matrix. It is secreted from odontoblasts, osteocytes, and osteoblasts, in order to bind hydroxyapatite and calcium during matrix mineralization [7]. It is one of the serological markers in the bone formation process. Numerous studies have shown increased OC levels in bone formation. However, increased OC level relates more to osteoid formation than matrix mineralization during bone formation [8–10]. Alkaline phosphatase (ALP) is a membrane-bound glycoprotein. Its function is catalyzing the hydrolysis of phosphate monoesters at a basic pH level. Bone-specific alkaline phosphatase (BALP) is known to be involved in bone calcification. It is secreted by osteoblasts to provide a high phosphate concentration at the osteoblast cell surface during bone mineralization [11].

The measurement of implant stability is based on the clinical, histological, biomechanical, and biochemical approaches. The resonance frequency analysis (RFA), a noninvasive clinical implant stability measurement, has been used in many studies. Meta-analysis of 47 studies has revealed a statistically significant correlation between RFA and insertion torque [12]. Numerous clinical studies have used the resonance frequency analysis technique on various implant designs to determine implant stability during the osseointegration period [13–20]. Evaluation of peri-implant crevicular fluid (PICF), a noninvasive, clinical, biochemical approach has been used to assess and to predict the peri-implant tissue loss [21, 22].

The purposes of this study were to examine the correlation between the stability of dental implants and bone formation markers during the healing period and to monitor the stability of dental implants during a 3-month period using the resonance frequency analysis method. The null hypothesis of the study is no correlation between the stability of dental implant and bone formation markers. Due to the three-thread-design of the implants, the authors also aim to measure the average implant stability quotient using RFA during the healing period.

## Methods

**Fig. 1:**
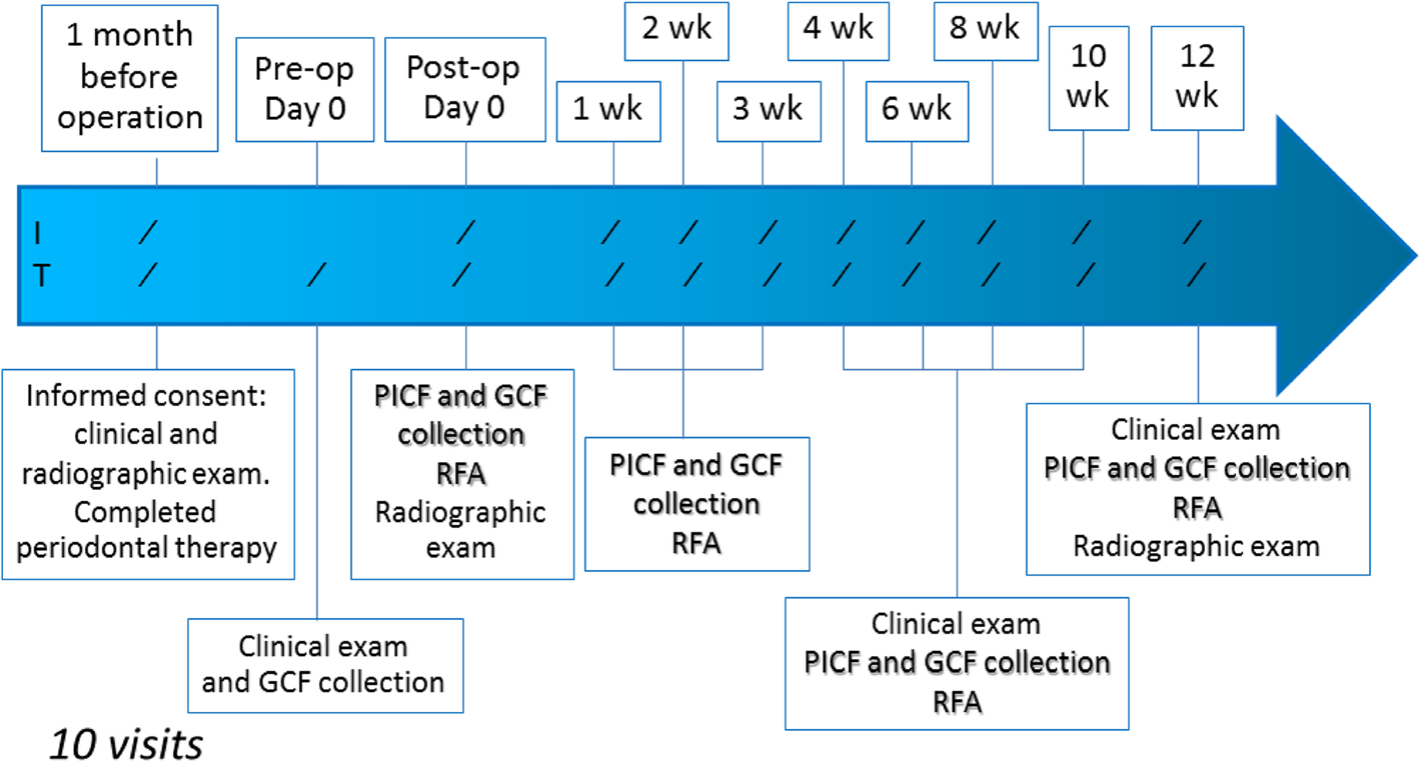
Timeline of the clinical study. *I*—implant site, *T*—contralateral posterior mandibular nonsurgical tooth

The study is a prospective clinical study during the 3-month healing period of implant. The study was approved by the Human Experimentation Committee, Faculty of Dentistry, Chiang Mai University. The study outline is shown in Fig. 1.

### Patients

Ten patients, who were partially edentulous in the mandibular posterior region for whom a single nonsubmerged implant was planned, participated in this study. All of them presented at the Center of Excellence for Dental Implantology, Faculty of Dentistry, Chiang Mai University, Thailand, between February and August 2015. The inclusion and exclusion criteria were shown in Table 1.

**Table 1:** Inclusion and exclusion criteria

Inclusion criteria	Exclusion criteria
Patients aged 25–65 years	Presence of periodontal disease or periapical lesions
Ability to participate in this study	History of bone augmentation at the implant site in the past 6 months
No systemic diseases (e.g., diabetes, osteoporosis, hypertension, etc.)	History of tooth extraction at the implant site in the past 6 months
Nonsmokers	A need for submersion of an implant
No contraindication for minor oral surgery	Requirement for grafting of bone or soft tissue at the time of implant insertion
No psychosis or psychiatric disorders	Pregnancy or positive to pregnancy test
No uncontrolled bleeding disorders	Postmenopausal women
No bisphosphonate, hormone replacement, or corticosteroid drug use	Physical disorders which would interfere with the ability to maintain oral health care
No anti-inflammatory drug use for 6 months before surgery	
Never received radiotherapy of the head and neck region	
Adequate oral hygiene (average modified sulcus bleeding index ≤1, average modified plaque index ≤1)	
Adequate bone volume to achieve the plan without other surgery (bone height ≥12 mm, bone width ≥7 mm)	
Existing healthy contralateral posterior mandibular tooth	

At the examination, the patients were informed about the study’s purposes, procedures, and possible risks and signed an informed consent. Scaling of all the teeth and oral hygiene instructions were performed for each patient.

### Surgical procedure

All surgical procedures were completed by the same implant surgeon. After clinical and radiographic examination, the latter using periapical, panoramic and cone beam computed tomography images, and 2 g of amoxicillin for antibiotic prophylaxis were administered to the patients 1 hour before surgery. One PW Plus® implant (PW Plus, Nakhon Pathom, Thailand) per patient was placed in the mature bone at bone level under local anesthesia (4% articaine with epinephrine 1:100,000). Midcrestal and sulcular incisions around the teeth were performed at the implant site. After full-thickness mucoperiosteal flap elevation, implant bed preparation was started by using custom surgical stents. Ten PW Plus® implants were placed according to the manufacturer’s recommendations. After implant insertion and stability measurement, the smooth (polished) healing abutment was screwed to the implant and the flaps were sutured using 4-0 polypropylene suture material. As post-operative instructions, the patients were asked to abstain from mechanical plaque control at the surgical sites, 0.12% chlorhexidine mouthwash was prescribed for microbial control, and 400 mg ibuprofen for analgesia.

### RFA assessments

At implant placement and after 1, 2, 3, 4, 6, 8, 10, and 12 weeks, RFA assessments were performed using the Osstell® ISQ (Integration Diagnostics AB, Goteborg, Sweden) according to the manufacturer’s instructions. A Smartpeg™ (type 47) (Integration Diagnostics AB) was screwed to the implant using a Smartpeg mount. After Smartpeg mount removal, the RFA assessment was performed with the measurement probe on the handheld Osstell® ISQ instrument. The measurement probe was held close to the top of the SmartPeg without touching it until the instrument emitted a beeping sound and the implant stability quotient (ISQ) value presented. Two measurements were performed, one from the buccal direction and one from the mesial direction. The two ISQ values were recorded.

### GCF and PICF sample collection

To observe the level of two bone formation biomarkers (alkaline phosphatase and osteocalcin) during the osseointegration period compared with control group using GCF from the first molar of the contralateral side of implant position, the sample collection of GCF was performed before the surgical procedure, immediately after the surgical operation and after 1, 2, 3, 4, 6, 8, 10, and 12 weeks. The PICF sample collection was performed immediately after the surgical operation and after 1, 2, 3, 4, 6, 8, 10, and 12 weeks by a single trained operator. PICF samples were collected from the buccal side of the implant healing abutment, and GCF samples were collected from the buccal side of the contralateral nonsurgical tooth. The GCF/PICF collecting techniques were modified from the method of Ciantar and Caruana [23]. Before collecting the samples, the sites were isolated with cotton rolls, supragingival plaque was removed by probe, and tissues were kept dry by gentle air flow. Paper strips (Periopaper, Oraflow, Smithtown, NY, USA) were inserted into the sulcus until resistance was felt and left in place for 30 s. The volume of fluid was immediately measured by Periotron 8000 (Periotron, Oraflow). The paper strips containing the GCF/PICF sample were kept in a 1.5-ml Eppendorf tube. The tube was labeled and kept on ice and then was transferred to −80 °C for storage until the analysis was performed.

### Sample preparation and analysis

GCF/PICF in the Periopaper strip was eluted by adding 320 μl quantity of phosphate-buffered saline (PBS) into the sample tube and incubated at 4 °C, overnight. The eluted protein solution from each gingival fluid sample was used for the biochemical analysis.

Total protein in the gingival fluid sample was measured by the Bradford analysis [24]. Briefly, a 10 μl volume of sample or protein standard (0–500 μg/ml) was added into each well of a 96-well microplate and then 200 μl of Bradford working reagent were added to each well. The microplate was shaken for 5 min. After that, the absorbance was measured at 620 nm. The concentrations of total protein in the samples were detected and calculated from a standard curve.

The level of OC was measured by using commercially available ELISA kits (Human Osteocalcin Quantikine ELISA Kit, R&D Systems, Inc., Minneapolis, MN, USA) according to the manufacturer’s instructions. A 100 μl volume of Assay Diluent RD1-117 (R&D Systems) was added into each well of the microplate and then 50 μl of standard (0–64 ng/ml) or sample was added to each well. The microplate was incubated for 2 h at room temperature on a horizontal orbital microplate shaker (Labnet International, Inc., Edison, NJ, USA) set at 500 rpm. After that, the solutions were aspirated and each well was washed with 400 μl of Wash Buffer (R&D Systems). This step was repeated three times for a total of four washes. Then, 200 μl of Human Osteocalcin Conjugate were added into each well, and the solutions were incubated for 2 h at room temperature on the shaker. After that, the solutions were aspirated and washed again. A 200 μl volume of substrate solution (tetramethylbenzidine) was added into each well, and the solutions were incubated for 30 min at room temperature on the bench-top. Next, 50 μl of stop solution (2 N sulfuric acid) were added into each well. The color in the wells changed from blue to yellow. After that, the color solution in each well was measured at 450/540 nm within 30 min. The OC level in each sample was calculated from a standard curve and normalized by total protein.

The level of the ALP activity was measured by colorimetric analysis. Briefly, 80 μl of sample solution were added into each well of a 96-well microplate, and then a mixture of 100 μl phosphate substrate solution (p-nitrophenyl phosphate) and 20 μl 1.5 M alkali buffer solution were added to each well. The microplate was incubated at 37 °C for 1 h. Next, 50 μl of stop solution (1 M NaOH) were added into each well. After that, the absorbance was measured at 620 nm. Known concentrations of ALP product (p-nitrophenol) were prepared with the dilutions ranging from 0 to 250 μM. The activity of ALP in the samples were calculated from a standard curve and normalized by total protein.

### Data analysis

Data were analyzed by using SPSS 17.0 software (SPSS Inc., Chicago, IL, USA). Repeated measures analysis of variance was performed to evaluate the change in the ISQ values at each measurement. The independent samples *t* test was used to investigate the differences in implant stability in the patients’ gender and bone quality. The Friedman test, followed by the Wilcoxon signed-rank test, was used to examine the differences in the crevicular fluid volume, the OC, or the ALP levels in each measurement. The Mann-Whitney *U* test was used to analyze the differences between the biomarker levels in the control and the test groups. The correlations between OC or ALP levels and ISQ values were calculated using the Pearson correlation coefficient. *P* values <0.05 were considered statistically significant.

## Results

Ten patients, seven females, and three males, aged 42.4 ± 11.99 years (range, 28 to 64 years), with either a first mandibular or second mandibular molar edentulous area, who required a single nonsubmerged implant participated in this study, as shown in Table 2. The implants used for all patients were 10 mm long and 5 mm in diameter. All patients completed the follow-up. None of the implants failed during the study period.

**Table 2:** Profile of patients

Patient no.	Age	Sex	Position^a^	Bone quality^b^
1	34	Female	46	3
2	38	Female	36	3
3	43	Female	37	3
4	64	Male	46	2
5	30	Female	47	3
6	48	Female	36	2
7	57	Male	36	3
8	28	Female	46	3
9	33	Male	46	2
10	49	Female	46	3

^a^FDI tooth-numbering system

^b^Lekholm et al. [36] bone classification

**Fig. 2:**
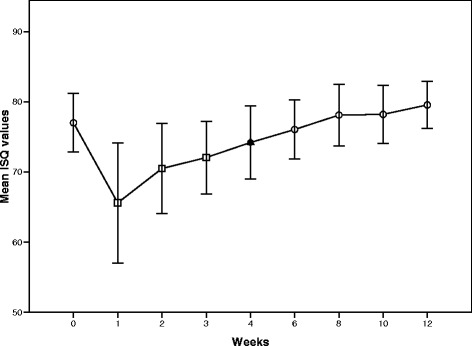
Change in the mean ISQ values over time. There was a statistically significant decrease in the mean ISQ values between 1 and 3 weeks (*P* < 0.05). The ISQ values recovered to the initial ISQ values at 4 weeks and slight increased at 6, 8, 10, and 12 weeks

The mean ISQ values were shown in Table 3. There was a statistically significant decrease in the mean ISQ values between 1 and 3 weeks (*P* < 0.05). The ISQ values recovered to the initial ISQ values at 4 weeks and slight increased at 6, 8, 10, and 12 weeks (Fig. 2). There was a significant increase in the mean ISQ values at 6, 8, 10, and 12 weeks when compared with 3 weeks. The mean lowest ISQ values, recorded at 1 week, were 65.6 ± 2.70. According to the independent samples *t* test, there were no statistically significant differences in the mean ISQ values with gender and bone quality.

**Table 3:** ISQ values according to gender and bone quality

Time	Day 0	1 week	2 weeks	3 weeks	4 weeks	6 weeks	8 weeks	10 weeks	12 weeks
Mean ISQ values	77.0 ± 1.32	65.6 ± 2.70^a^	70.5 ± 2.03^a^	72.1 ± 1.64^a^	74.2 ± 1.65	76.1 ± 1.33	78.1 ± 1.38	78.2 ± 1.32	79.6 ± 1.06
Gender									
Male (*n* = 3)	77.0 ± 0.58	59.7 ± 4.62	71.7 ± 3.24	75.0 ± 1.89	75.3 ± 3.92	77.8 ± 2.17	80.2 ± 3.44	78.2 ± 3.35	79.5 ± 2.78
Female (*n* = 7)	77.0 ± 1.92	68.1 ± 3.00	70.0 ± 2.68	70.8 ± 2.08	73.7 ± 1.88	75.3 ± 1.67	77.2 ± 1.40	78.2 ± 1.46	79.6 ± 1.13
Bone quality^a^									
Type 2 (*n* = 3)	79.3 ± 1.86	60.7 ± 3.77	67.7 ± 4.60	74.5 ± 1.89	73.3 ± 3.32	76.0 ± 2.02	78.7 ± 3.37	77.3 ± 3.09	78.2 ± 2.20
Type 3 (*n* = 7)	76.0 ± 1.64	67.7 ± 3.34	71.7 ± 2.23	71.0 ± 2.15	74.6 ± 2.05	76.1 ± 1.79	77.9 ± 1.56	78.6 ± 1.51	80.1 ± 1.22

^a^Lekholm et al. [36] bone classification

**Fig. 3:**
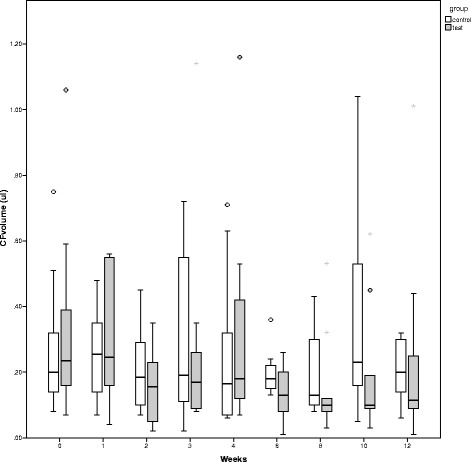
Change in the median values of the GCF (control group) and PICF (test group) volume over time. In the test group, the PICF volume continuously decreased with time (**a**). There were no significant differences in the median values of the crevicular fluid volume in either the control group or the test groups at any measurement (**b**)

The median values of the GCF and PICF volume are shown in Table 4. At the implant site (test group), the PICF volume continuously decreased with time (Fig. 3). However, according to the Friedman test, there were no significant differences in the median values of the crevicular fluid volume in the control or the test group at each measurement.

**Table 4:** Crevicular fluid volume

Time	Day 0	1 week	2 weeks	3 weeks	4 weeks	6 weeks	8 weeks	10 weeks	12 weeks
Median (interquartile range)									
CF volume (μl)									
Tooth (control)	0.20 (0.23)	0.26 (0.25)	0.19 (0.20)	0.19 (0.50)	0.17 (0.33)	0.18 (0.08)	0.13 (0.24)	0.23 (0.42)	0.20 (0.17)
Implant (test)	0.26 (0.30)	0.25 (0.41)	0.16 (0.21)	0.17 (0.19)	0.18 (0.33)	0.13 (0.14)	0.10 (0.09)	0.10 (0.17)	0.12 (0.21)

**Fig. 4:**
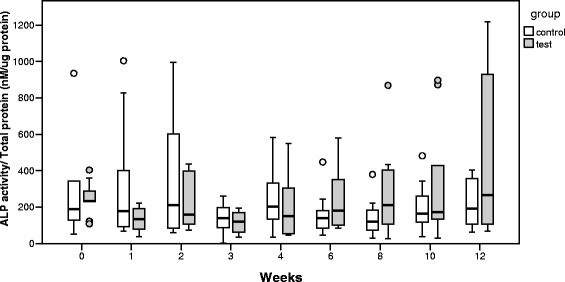
Change in the median values of the ALP level over time. In the test group, the ALP level decreased at 1–4 weeks and then increased at 6, 8, 10, and 12 weeks. There was no statistically significant difference in the ALP level in either the control or the test groups at any measurement

Both ALP and OC molecules were detectable in GCF and PICF samples. The median values of the ALP and OC levels are shown in Table 5. In the control group, there was no significant difference in the ALP or the OC levels at each measurement. In the test group (implant site), the ALP level decreased at 1–4 weeks and then increased at 6, 8, 10, and 12 weeks (Fig. 4). However, according to the Friedman test, there was no statistically significant difference in the ALP level of the test group at each measurement. Furthermore, according to the Mann-Whitney *U* test, there was no statistically significant difference in the ALP level between the control and the test group at each measurement.

**Table 5:** Crevicular fluid ALP and OC levels

Time	Day 0	1 week	2 weeks	3 weeks	4 weeks	6 weeks	8 weeks	10 weeks	12 weeks
Median (interquartile range)									
ALP level									
(nM/μg protein)									
Tooth (control)	175 (215)	203 (308)	148 (269)	143 (112)	266 (427)	145 (96)	181 (148)	191 (263)	107 (128)
Implant (test)	230 (238)	139 (139)	157 (293)	108 (134)	166 (434)	179 (251)	147 (296)	157 (201)	151 (968)
OC level									
(pg/μg protein)									
Tooth (control)	30 (42)	19 (56)	40 (92)	31 (34)	29 (48)	23 (31)	50 (49)	40 (78)	28 (47)
Implant (test)	7 (15)	16 (69)	15 (81)	68 (46)	37 (79)	52 (105)	94 (292)	91 (87)	59 (94)

**Fig. 5:**
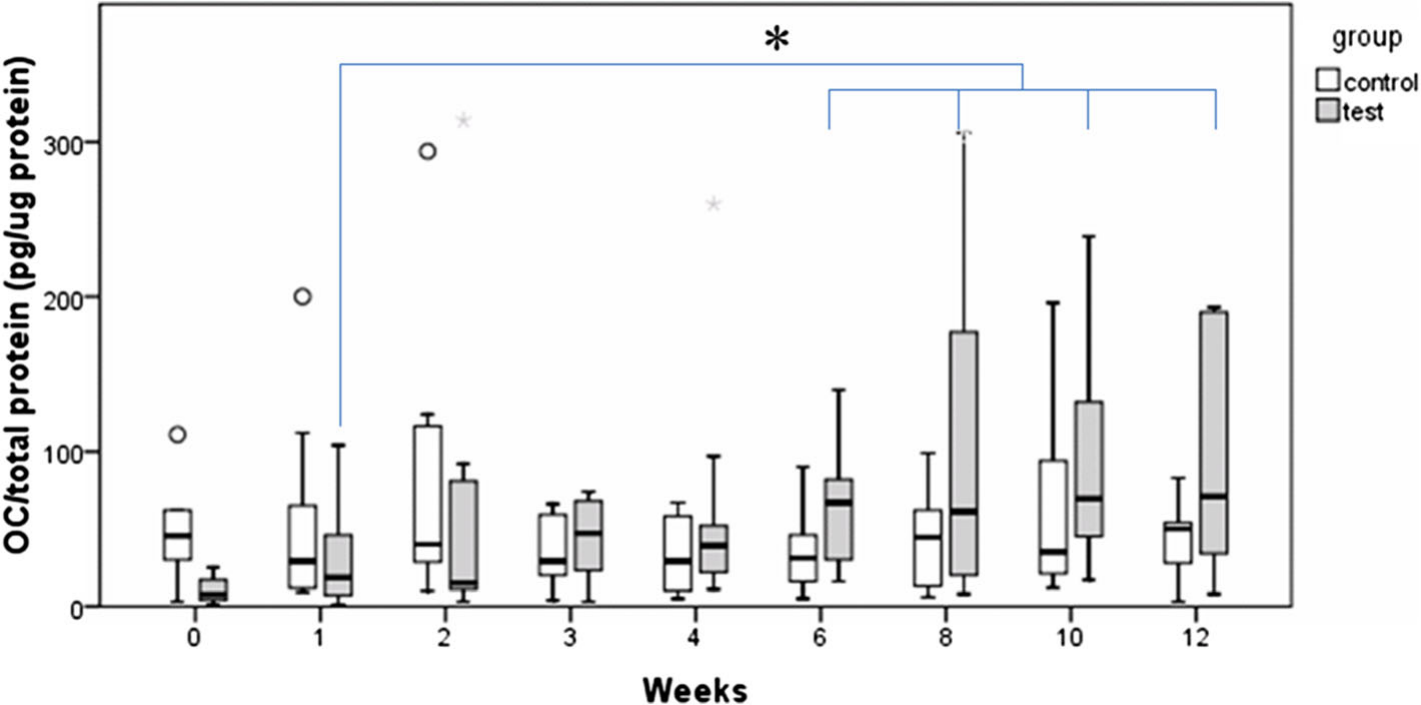
Change in the median values of the OC level over time. In the test group, the OC level continuously increased with time. There was a statistically significant increase in the OC level at 6, 8, 10, and 12 weeks when compared with 1 week (*P* < 0.05)

At the implant site, the OC level continuously increased with time. According to the Friedman followed by Wilcoxon signed-rank tests, there was statistically significant increase in the OC level at 6, 8, 10, and 12 weeks when compared with 1 week (*P* < 0.05, Fig. 5). Furthermore, according to the Mann-Whitney *U* test, there was a statistically significant difference in the OC level between the control and the test group at 6, 8, 10, and 12 weeks.

**Fig. 6:**
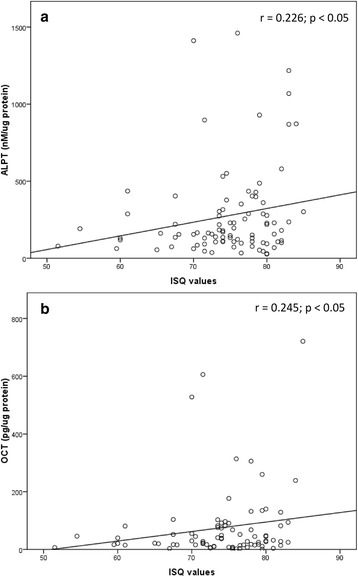
There were weakly significant and positive correlations between the ALP or OC levels and ISQ values at all measurements from week 1 to week 12. At the implant site, the ALP levels in nM/μg protein (**a**) or the OC levels in pg/μg protein (**b**) were associated with ISQ values

**Fig. 7:**
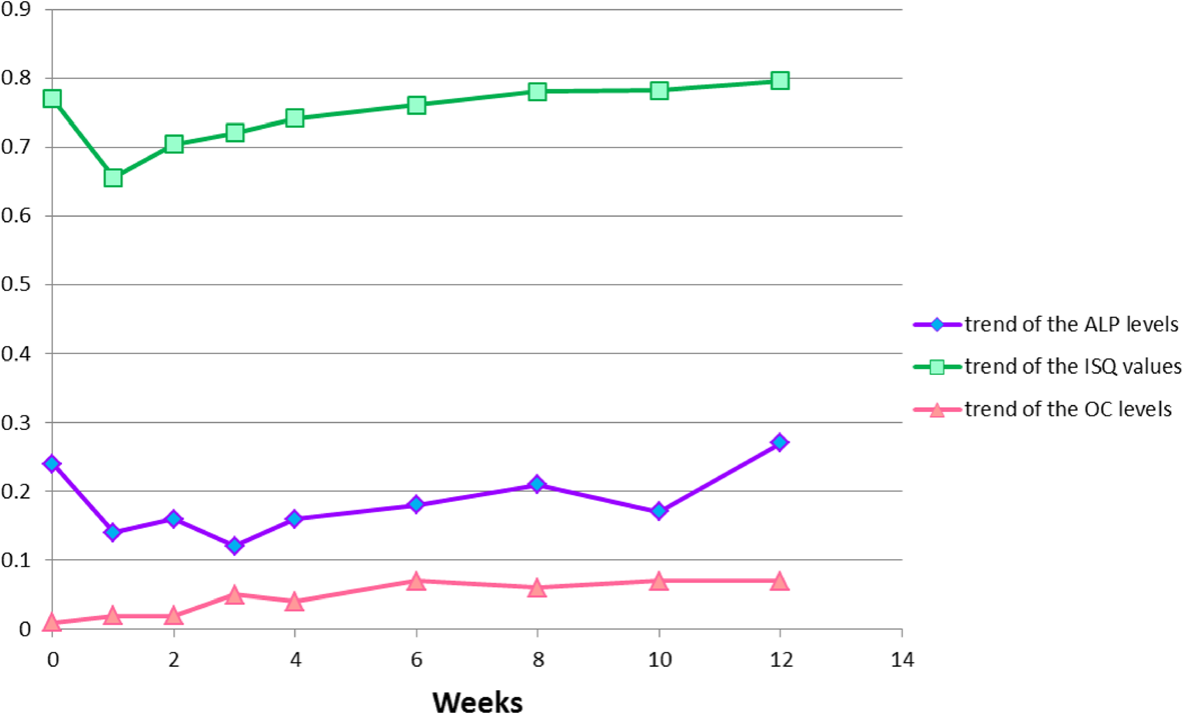
Comparison between the trend of the biomarker levels and the trend of the ISQ values over time

**Fig. 8:**
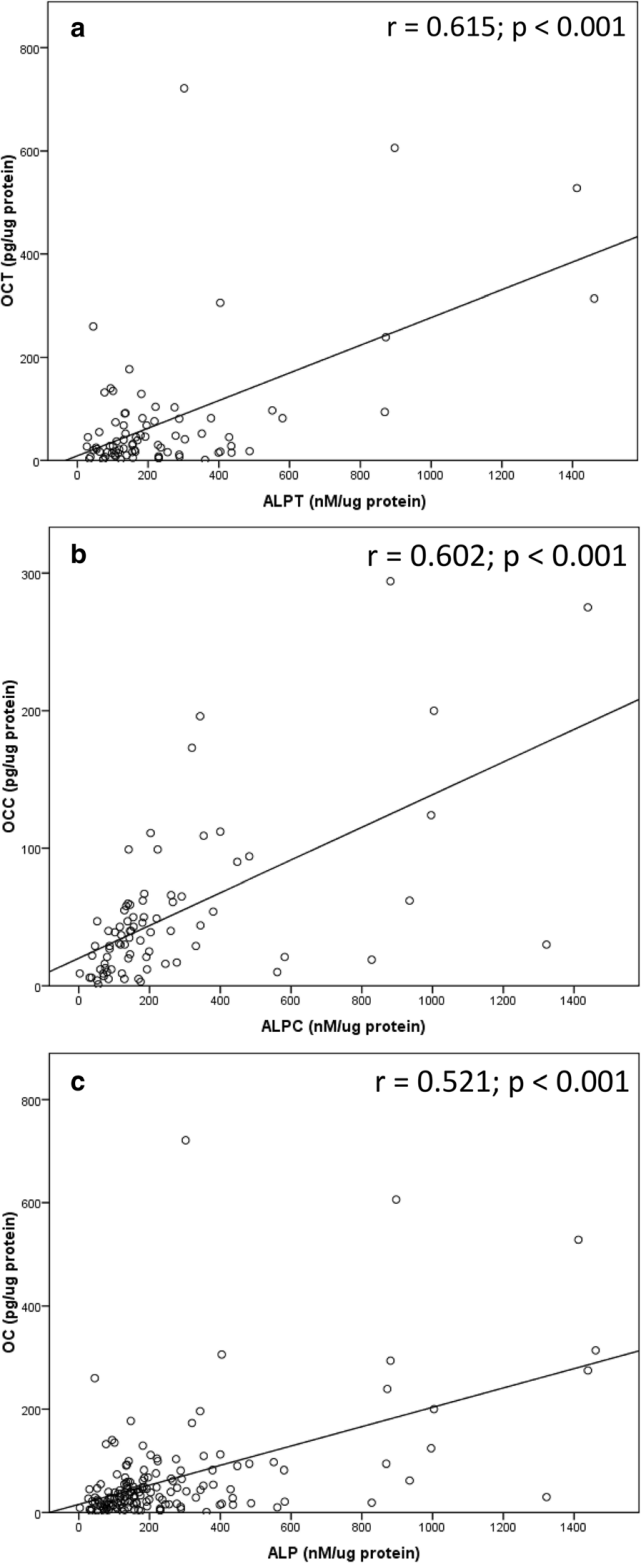
There were moderately significant and positive correlations between the ALP levels and OC levels at all measurements from week 1 to week 12. The OC levels in pg/μg protein were associated with the ALP levels in nM/μg protein at the implant site (**a**), control site (**b**), and pooled samples of the control and implant sites (**c**)

Correlations between ALP or OC levels and ISQ values were examined, as shown in Figs. 6 and 7. During 1–12 weeks, there was a statistically significant correlation between ALP levels at the implant site and ISQ values (*r* = 0.226; *P* < 0.05). There was a statistically significant correlation between OC levels at the implant site and ISQ values at 1–12 weeks (*r* = 0.245; *P* < 0.05). Correlations between ALP and OC levels are shown in Fig. 8. At all measurements from week 1 to week 12, the ALP levels were moderately correlated with the OC levels at the implant site, the control site, and the pooled samples of the control and implant sites (*r* = 0.615, 0.602, and 0.521, respectively, *P* < 0.001).

## Discussion

The results of this study show that, at the time of implantation, the ISQ values ranged between 67.5 and 83. The mean initial ISQ value was 77.0 ± 1.32. These findings are in harmony with those of previous studies [16, 18]. Tallarico et al. (2011) reported that the macro-design of dental implant affects the primary anchorage [16]. They suggested that the high initial ISQ value is a factor in making decisions about immediate loading on dental implants. The three-thread-design implants used in this study showed a high primary stability (ISQ value ≥65 to 70).

In the first 3 weeks, the mean ISQ values decreased and then increased and reached the initial values in the fourth week. The values then constantly increased in the following weeks. The lowest ISQ value in an implant was 51.5, found in the first week, and the highest value was 84, found in the tenth week. These results are in harmony with the findings of other clinical studies in that the ISQ values decreased within the first 3 weeks after implantation, and then increased and reached the initial values in the following weeks [17, 25]. These findings advise the presence of an interval between primary and secondary stability. An animal study, using a bone chamber model, which investigated the different phases of wound healing surrounding dental implants, showed that in areas of primary stability at the pitch region of the implant, osseointegration occurred after a resorptive process [26]. Thus, the primary mechanical stability was destroyed for a short period of time. However, the results of another study showed no difference in the ISQ values over 12 weeks [18].

In considering the median values of the crevicular fluid volume, it was demonstrated that the PICF volume continuously decreased with time. This finding is in harmony with the result of a recent clinical study that investigated the differences between peri-implant and periodontal wound healing [27]. That study showed that PICF volume decreased significantly from week 1 to week 3. Alteration of the gingival fluid volume and contents occur according to the condition of the tissues surrounding the teeth. The presence of inflammation increases the gingival fluid volume. Also, changes in peri-implant crevicular fluid contents and volume result from the condition of the peri-implant tissue.

The main sources of ALP in GCF are neutrophils, bacteria within dental plaque, fibroblasts, and osteoblasts [28]. Plagnat et al. [29] suggested that longitudinal monitoring of ALP in PICF might confirm its possible use as a marker of implant failure. Considering the change in the median values of the ALP level over time, in the test group, the ALP level decreased at 1–4 weeks and then increased at 6, 8, 10, and 12 weeks. These results are similar to those of a previously reported animal study of gene expression of ALP during the osseointegration period [30]. However, no significant differences in the ALP level were noted over time. This may be due to the variation of ALP sources in PICF and/or the small sample size at each measurement in this study.

Osteocalcin or bone gamma-carboxyglutamate protein is a noncollagenous protein in the bone matrix. It is a small (6000 Da) polypeptide. Monjo et al. [30] studied 372 implants in 93 rabbits, and suggested that osteocalcin is the best biological marker for implant osseointegration after 4-week healing periods. In our study, there were statistically significant increases in the OC level at 6, 8, 10, and 12 weeks when compared with 1 week. These results are similar to those of previous clinical studies. Slotte et al. [31] studied gene expression of bone healing in PICF using a quantitative real-time PCR technique [31]. Their pilot study demonstrated that gene expression for OC was high 7 weeks after implant placement. Prati et al. [32] studied the release of bone markers during the osseointegration period using the Luminex assay. They revealed that OC level was high 8 weeks after implant placement in their nonloaded group.

Regarding the relationship between the ISQ values and ALP or OC levels, although the ISQ values were weakly correlated with the bone markers (*r* = 0.226 for ALP level and *r* = 0.245 for OC level, *P* = 0.05), there were significant and positive correlations between the ISQ values and ALP or OC levels at all measurements from week 1 to week 12. These results are in harmony with the findings of a previous clinical study that investigated gene expression of bone markers in PICF [31]. That pilot study showed that ALP gene expression was correlated with RFA at week 4 (*r* = 0.44, *P* = 0.03), and OC gene expression was correlated with RFA at week 2 (*r* = 0.5, *P* = 0.04). To our knowledge, correlations of the ISQ value with the bone markers which were detected at protein level in PICF are reported for the first time.

Vogel and Marcotte [33] suggested that the correlation between mRNA and protein quantities is approximately 40%. As there are many mechanisms between transcription and translation, especially in human cell and protein stability, at the gene expression level, the transcription data is beneficial in making decisions about molecular candidates for future studies at the protein level. The colorimetric assay and ELISA techniques utilized in this study have many advantages, such as sensitivity, specificity, speed, and compatibility with standard clinical laboratory equipment [34]. These techniques can be routinely applied in clinical situations. Although the high sensitivity and specificity of the quantitative, real-time, polymerase chain reaction are justified [35], the limitations of this technique are the sensitive nature of the work and the cost of the chemicals and equipment.

## Conclusions

Within the limitations of this study and cautious interpretation due to small number of implants/patients, the ISQ values were weakly correlated with both ALP and OC molecules in PICF during the healing period. The results also show that osteocalcin may be used as a biological marker for monitoring implant healing at 6, 8, 10, and 12 weeks after implant placement. The ISQ values showed high stability of dental implants used over the healing period.
